# Transient spontaneous remission in congenital *MLL-AF10* rearranged acute myeloid leukemia presenting with cardiorespiratory failure and meconium ileus

**DOI:** 10.1186/s40348-016-0061-7

**Published:** 2016-08-29

**Authors:** Tobias Gyárfás, Juergen Wintgens, Wolfgang Biskup, Ilske Oschlies, Wolfram Klapper, Reiner Siebert, Susanne Bens, Claudia Haferlach, Roland Meisel, Michaela Kuhlen, Arndt Borkhardt

**Affiliations:** 1 Medical Faculty, Department of General Pediatrics, Neonatology and Pediatric Cardiology, Centre for Child and Adolescent Health, University of Duesseldorf, Duesseldorf, Germany; 2 Department of Neonatology, Staedtische Kliniken Moenchengladbach, Elisabeth Krankenhaus Rheydt, Rheydt, Germany; 3 Department of Pathology, Christian-Albrechts-University Kiel, Kiel, Germany; 4 Institute of Human Genetics, Christian-Albrechts-University Kiel, Kiel, Germany; 5 MLL Munich Leukemia Laboratory, Munich, Germany; 6 Department of Pediatric Oncology, Hematology and Clinical Immunology, University Children’s Hospital, Medical Faculty, Heinrich Heine University, Moorenstr. 5, 40225 Duesseldorf, Germany

**Keywords:** Acute myeloid leukemia, Congenital, Meconium ileus, Cardiorespiratory failure, Spontaneous remission, Case report

## Abstract

**Background:**

Neonatal leukemia is a rare disease with an estimated prevalence of about one to five in a million neonates. The majority being acute myeloid leukemia (AML), neonatal leukemia can present with a variety of symptoms including hyperleucocytosis, cytopenia, hepatosplenomegaly, and skin infiltrates. Chromosomal rearrangements including mixed lineage leukemia (MLL) translocations are common in neonatal AML.

**Case presentation:**

A female neonate born at 34 weeks gestation presented with cardiorespiratory failure, hepatosplenomegaly, pancytopenia, and coagulopathy. She required intensive care treatment including mechanical ventilation, high-dose catecholamine therapy, and multiple transfusions. Small intestinal biopsy obtained during laparotomy for meconium ileus revealed an infiltrate by an undifferentiated monoblastic, MLL-rearranged leukemia. No other manifestations of leukemia could be detected. After spontaneous clinical remission, lasting 5 months without any specific treatment, the patient presented with leukemia cutis and full-blown monoblastic leukemia. MLL-AF10-rearranged AML could be re-diagnosed and successfully treated with chemotherapy and hematopoietic stem cell transplantation.

**Conclusions:**

Our patient exhibited a unique manifestation of neonatal MLL-AF10 rearranged AML with cardiorespiratory failure and intestinal infiltration. It highlights the importance of leukemia in the differential diagnosis of neonatal distress, congenital hematological abnormalities, and skin lesions.

## Correspondence/findings

### Background

Acute myeloid leukemia (AML) accounts for about 18 % of pediatric leukemias [1]. Disease onset during the neonatal period is extremely rare, with an estimated prevalence of one to five in a million neonates. The majority of neonatal leukemias are AML, and the most common type is monoblastic leukemia (FAB M5). Typical clinical features are skin infiltrations, hepatosplenomegaly, and cardiac failure. Prenatal symptoms including non-immune hydrops and polyhydramnios illustrate the in utero origin of the disease [2]. Like in infant leukemias, chromosomal rearrangements are regularly found in neonatal leukemias [2, 3] with chromosome 11q23/*MLL* rearrangements being by far the most common ones. [4].

### Case report

We present the case of a 14-month-old girl from birth until today. Pregnancy was uneventful until 34 weeks gestation, when polyhydramnios, pathological cerebral Doppler flow, pleural effusions, and pathological cardiotocography led to performing a cesarean section. The girl was born pale, with no muscle tone, no spontaneous breathing, and bradycardia. The APGAR score was 2/6/7 at 1/5/10 min. Persisting respiratory insufficiency required maximally invasive treatment including the administration of endotracheal surfactant, the use of a high-frequency oscillation ventilator and nitrogen monoxide to maintain sufficient oxygenation. Continuous dobutamine and adrenaline infusions were necessary due to severe circulatory deterioration starting immediately after birth. Echocardiogram revealed pulmonary hypertension and compromised right ventricular function. Massive hepatosplenomegaly was detectable on abdominal ultrasound.

An initial hemoglobin value of 6.3 g/dl led to an emergency erythrocyte transfusion, and further erythrocyte transfusions were necessary in the first weeks of life. Multiple petechiae and suggilations were present at birth, thrombocytopenia and disseminated intravascular coagulopathy with unmeasurable thrombin time repeatedly required multiple thrombocyte concentrate and fresh-frozen plasma transfusions. An initial leukocyte count of 45.000/μl rapidly dropped to a minimum of 600/μl.

The etiology of the clinical condition with cardiorespiratory failure, pancytopenia, and disseminated intravascular coagulopathy together with skin hemorrhages and hepatosplenomegaly was at first elusive. Ganciclovir was initiated for suspected cytomegalovirus infection but withdrawn following negative results. Given the possibility of neonatal sepsis, various antibiotics and fluconazole were administered, but inflammatory values did not markedly increase and microbiological cultures and extensive viral PCR studies yielded exclusively negative results. Peripheral blood smears from the fourth day of life revealed immature monocytic cells, and bone marrow puncture was found unrepresentative without evidence of blastic cells. Additional flow cytometry was unremarkable. Genetic predisposition for hemophagocytic lymphohistiocytosis was excluded.

The girl’s clinical condition remained highly instable, when on the tenth day of life the assessment of acute abdomen required explorative laparotomy. During surgery, a meconium ileus was found that could only be resolved by establishment of an ileostomy. Because of a massively distended abdomen, abdominal closure could only be realized by placement of a patch. Buccal mucosa and ileum biopsies were undertaken to exclude neonatal hemochromatosis.

**Fig. 1:**
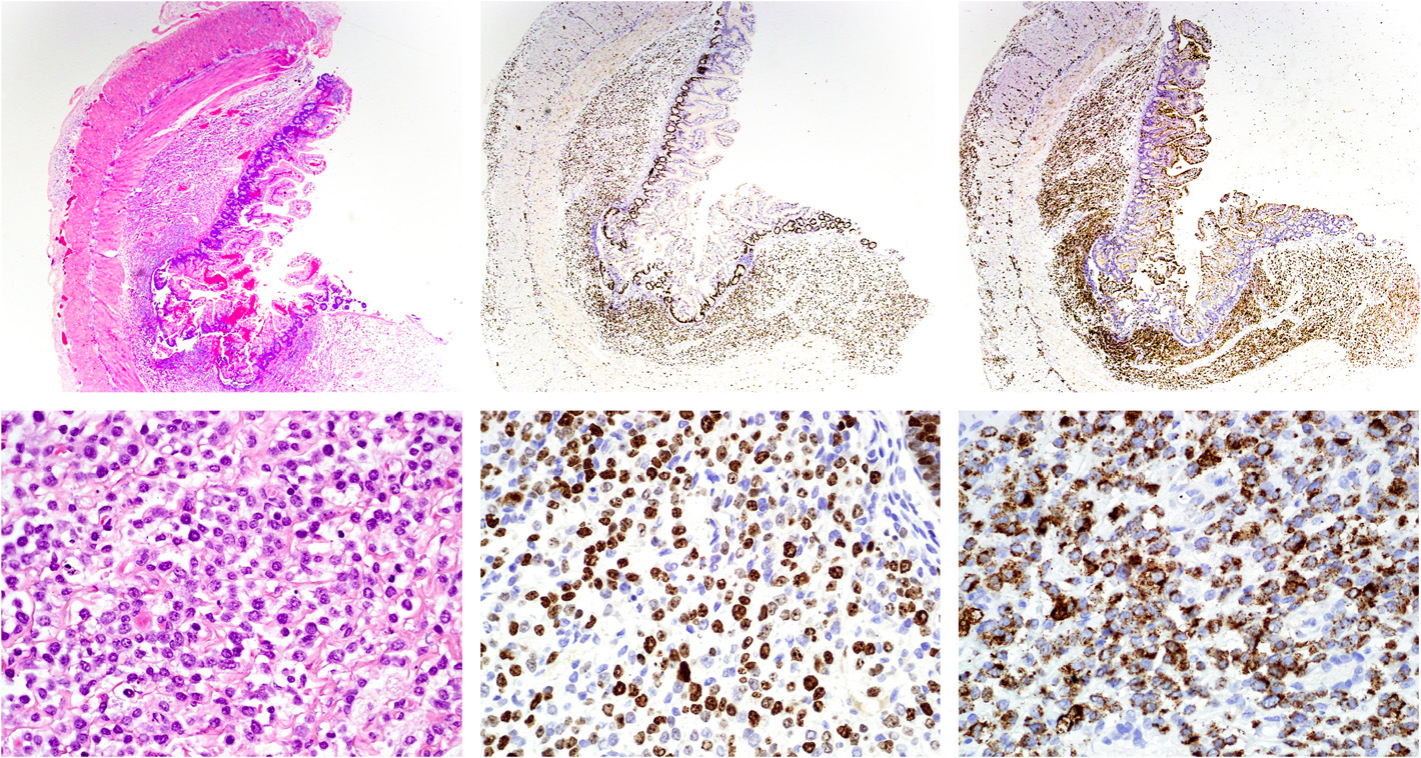
Submucosal infiltrate of polymorphic, atypical cells in the terminal ileum. *Top row*: ×25 magnification, bottom row: ×400 magnification. *Left*: hematoxylin and eosin (H&E) stain. *Middle*: KI67 staining shows a proliferation rate of 80 %. *Right*: the majority of cells display positive staining for the monocyctic marker CD68. A clonal *MLL* breakpoint in 11q23 was detected by FISH on paraffin embedded tissue-slides in the cells of the intestinal infiltrate. The population was classified as an extramedullary manifestation of monoblastic acute myeloid leukemia (AML FAB M5)

Unexpectedly, pathological examination revealed diffuse submucosal infiltration by polymorphous, atypical cells suspicious of Langerhans cell histiocytosis (LCH). Single-shot treatment with vincristine (0.05 mg/kg) and prednisolone (1.5 mg/kg/d) for suspected LCH was immediately commenced. Five days later, the LCH diagnosis was rejected by reference pathology evaluation and reclassified as completely undifferentiated blastic transmural infiltration with diffuse growth pattern, positive staining for CD68, partial expression of S100, negative staining for langerin, lysozyme, CD1a, myeloperoxidase, CD34 ,and CD30, and an increased proliferation rate of 80 % (MIB1) (Fig. 1).

To further classify the blastic infiltration, additional immuno-histochemical staining was performed and revealed partial expression of CD163, negativity for CD56, CD117, CD3, CD79a, desmin, NB84, pan-cytokeratin, synaptophysin, and TdT, and preserved nuclear expression of INI1, thus being consistent with an undifferentiated leukemia with partial monoblastic differentiation. Further, a chromosomal breakpoint at the mixed lineage leukemia (*MLL*) locus 11q23 could be detected by fluorescence in situ hybridization (FISH, LSI MLL Dual Color, Break Apart Probe, Abbott) substantiating the diagnosis of an undifferentiated, *MLL* positive leukemia. Two subsequent bone marrow punctures at the age of 3 and 5 weeks did not show any signs of leukemia, and no *MLL* rearrangement could be detected by FISH (Table 1).

**Table 1:** Results of molecular studies on *MLL* rearrangement

Sample type	Obtained at age	Method	Analysis	Result
Ileum biopsy	10 days	FISH	*MLL* rearrangement	Positive
Bone marrow	3 weeks	FISH	*MLL* rearrangement	Not feasible
		Nested PCR	*MLL-AF10*	Positive (in retrospect)
Bone marrow	5 weeks	FISH	*MLL* rearrangement	Negative
		Nested PCR	*MLL-AF10*	Positive (in retrospect)
Peripheral blood	5 months	FISH	*MLL* rearrangement	Positive
		RT-PCR^a^	*MLL-AF10*	Positive
Bone marrow	5 months	RT-PCR^a^	*MLL-AF10*	Positive

^a^Quantitative real time PCR

Subsequently, leukocyte, erythrocyte, and thrombocyte counts recovered spontaneously, accompanied by gradual clinical improvement including complete weaning from ventilator support and reversal of ileostomy. The patient was discharged at 6 weeks of age and a weight of 2900 g in excellent clinical condition.

**Fig. 2:**
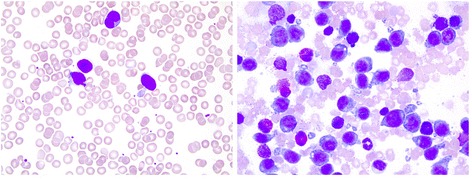
Monoblastic leukemia (AML FAB M5) (×63 magnification) diagnosed from peripheral blood (*left*) and bone marrow (*right*) at the age of 5 months

Subsequent clinical follow-up displayed regular weight gain (weight at birth 3000 g, at the age of 6 weeks 2900 g, at 3 months 4300 g) and development and routine hematological controls remained unremarkable. Five months later, while on treatment for hemangioma with propranolol, suspicious, blue-berry muffin-like skin lesions were noted on clinical examination. Leukocyte count was 28.500/μl, and thrombocyte and erythrocyte counts were normal. Monoblastic leukemia (AML FAB M5) was diagnosed from peripheral blood and bone marrow (Fig. 2). An *MLL-AF10* fusion transcript was detected by real-time and nested PCR. Retrospective analyses (by nested PCR) at the time point, when bone marrow sampled in the third week of life was *MLL*-rearrangement-negative by FISH, showed that the *MLL-AF10* fusion transcript was already detectable back then, 4 months prior to the definitive diagnosis of AML in the bone marrow (Table 1).

Chemotherapy was initiated according to the German AML-BFM 2012 study protocol [5]. Due to the *MLL-AF10* rearrangement, the patient was classified as “high risk” and consequently qualified for hematopoietic stem cell transplantation (hSCT). The patient received one block of induction and two blocks of consolidation chemotherapy. Subsequently, the patient underwent hSCT from an unrelated, HLA-matched donor. The conditioning regimen consisted of busilvex, cyclophosphamide, melphalan, and anti-thymocyte globuline (ATG). Cyclosporin A and methotrexate were administered as prophylaxis against Graft versus Host Disease (GvHD). Leukocyte engraftment was detected at day 19, granulocyte engraftment at day 23 post transplantation. Chemotherapy and stem cell transplantation were well tolerated under supportive care. The girl was discharged at day +60. Apart from partial compromise of renal function, no significant complications occurred. Until now, the patient is well, relapse-free, and showing no signs of GvHD 9 months after transplantation. Donor chimerism is 100 %.

### Discussion

Neonatal leukemia is extremely rare. It accounts for about 1 % of pediatric leukemias and has an estimated prevalence of one to five in a million neonates. The disease was already known in the early twentieth century [6]. Neonatal AML shows similarities to transient myeloproliferative disorder (TMD), an abnormal proliferation of myeloid blasts observed in Down’s syndrome that usually resolves without therapy [7]. A few cases with spontaneous remission, which in one case was transient, mimicking TMD of Down syndrome, have been described in neonates with normal karyotypes [2, 8, 9]. Isolated leukemia cutis has also been described to disappear spontaneously [10, 11]. However, to the best of our knowledge, no case of a preterm baby with persistent pulmonary hypertension (PPHN) and cardiorespiratory failure requiring NO-based artificial ventilation and high-dose catecholamines could successfully be rescued by combined efforts of intensive care, chemotherapy, and stem cell transplantation.

The clinical picture of neonatal leukemia is variable. Common symptoms include leukocytosis, cytopenia, hepatosplenomegaly, and skin infiltrations. The latter are known as leukemia cutis, appear in about 60 %, and are the first symptom in about one half of neonatal leukemia cases. Leukemia cutis, presenting as firm nodules, papules or plaques of red, blue or purple color, is a differential diagnosis of the so-called blueberry muffin baby. This syndrome has a diversified differential, ranging from hemato-oncological disorders like Langerhans cell histiocytosis, neuroblastoma, or rhabdomyosarcoma to infectious diseases like rubella, cytomegalovirus infection, or toxoplasmosis to hemolytic disease and blue rubber bleb nevus syndrome [12, 13]. Leukemia cutis has been described as an exclusive manifestation of AML without bone marrow disease [14], as well as appearing prior to or simultaneously with bone marrow involvement [13, 15]. Our patient presented with severe bruising at birth, probably due to thrombocytopenia and coagulopathy. Months later after presumed relapse of the so far not unequivocally diagnosed AML, she primarily presented with skin infiltrations.

As a hematological malignancy, leukemia is essentially a distributed disease, but primary presentation as extramedullary infiltration, also called myeloid sarcoma, is a rarity in AML. Pediatric myeloid sarcoma has a prevalence of about 0.7 in one million children and can progress to AML [16, 17]. Infiltration of the gastrointestinal tract, including the ileum, has been reported in AML [18, 19]. In our patient, acute meconium ileus appeared on the tenth day of life. An extensive infiltration of monoblasts was found as the probable cause of the mechanical ileus. To our knowledge, such a condition has also not yet been described in neonatal leukemia.

Chromosome 11q23 rearrangements involving the *MLL* gene locus are common in infant leukemia; they are the most frequent finding in neonatal AML [2]. Specifically, our patient had an *MLL-AF10* fusion transcript, which is rather rarely found among the large variety of the various *MLL* rearrangements [20]. In the AML-BFM 2012 protocol, *MLL-AF10* positive patients are considered “high risk” and recruited to a treatment protocol including hSCT. In our patient, a *MLL* rearrangement was first diagnosed from the intestinal infiltrate found in the terminal ileum and a *MLL-AF10* fusion transcript was first detected at 5 months of age. In retrospect, its presence could then be verified from the bone marrow obtained at the age of 3 and 5 weeks by a sensitive PCR assay.

As from the first days of life there is no more material available, it remains somehow unclear of what extent the leukemia initially presented and whether the cytopenia was due to bone marrow involvement or whether it was associated to cytokine release and/or coagulopathy. In the following weeks, the clinical condition improved and no signs of leukemia could be detected. It cannot be ruled out with certainty that regression of the leukemia was not spontaneous but caused by a single dose of vincristine and prednisolone treatment; however, given the prolonged recovery, the latter seems unlikely. Thus, other reasons for spontaneous regression of this aggressive type of leukemia, such as infection or disease related cytokine release, should be considered.

Taken together, these findings indicate that our patient had a neonatal, *MLL-AF10* positive AML with spontaneous transient regression that later reappeared as leukemia cutis and bone marrow disease.

### Conclusion

We present a unique manifestation of neonatal *MLL-AF10* positive AML with cardiorespiratory failure at birth, intestinal infiltration, and skin involvement. After a transient spontaneous regression, the patient was successfully treated by chemotherapy and hSCT and is alive and well.

### Abbreviations

AF10, ALL1-fused gene from chromosome 10 protein; AML, acute myeloid leukemia; ATG, anti-thymocyte globuline; BFM, Berlin Frankfurt Münster; CD, cluster of differentiation; FAB, French-American-British; FISH, fluorescence in situ hybridization; GvHD, Graft versus Host Disease; hSCT, hematopoietic stem cell transplantation; LCH, Langerhans cell histiocytosis; MLL, mixed lineage leukemia; PCR, polymerase chain reaction; PPNH, persistent pulmonary hypertension; RT-PCR, real-time PCR; TMD, transient myeloproliferative disorder
